# Pharmacological interventions for social cognitive impairments in schizophrenia: A protocol for a systematic review and network meta-analysis

**DOI:** 10.3389/fpsyg.2022.878829

**Published:** 2022-08-03

**Authors:** Yuji Yamada, Ryo Okubo, Hisateru Tachimori, Takashi Uchino, Ryotaro Kubota, Hiroki Okano, Shuhei Ishikawa, Toru Horinouchi, Keisuke Takanobu, Ryo Sawagashira, Yumi Hasegawa, Yohei Sasaki, Motohiro Nishiuchi, Takahiro Kawashima, Yui Tomo, Naoki Hashimoto, Satoru Ikezawa, Takahiro Nemoto, Norio Watanabe, Tomiki Sumiyoshi

**Affiliations:** ^1^Department of Psychiatry, National Center Hospital, National Center of Neurology and Psychiatry, Tokyo, Japan; ^2^Clinical Research & Education Promotion Division, National Center of Neurology and Psychiatry, Tokyo, Japan; ^3^Endowed Course for Health System Innovation, Keio University School of Medicine, Tokyo, Japan; ^4^Department of Neuropsychiatry, Faculty of Medicine, Toho University, Tokyo, Japan; ^5^Department of Neuropsychiatry, Graduate School of Medicine, University of Yamanashi, Yamanashi, Japan; ^6^Department of Psychiatry, Hokkaido University Hospital, Sapporo, Japan; ^7^Department of Psychiatry, Graduate School of Medicine, Hokkaido University, Sapporo, Japan; ^8^Department of Preventive Intervention for Psychiatric Disorders, National Institute of Mental Health, National Center of Neurology and Psychiatry, Tokyo, Japan; ^9^Graduate School of Human and Social Sciences, Musashino University, Tokyo, Japan; ^10^Endowed Institute for Empowering Gifted Minds, Graduate School of Arts and Sciences, The University of Tokyo, Tokyo, Japan; ^11^Department of Psychiatry, Soseikai General Hospital, Kyoto, Japan

**Keywords:** pharmacotherapy, schizophrenia, social cognition, systematic review, network meta-analysis

## Abstract

**Background:**

Social cognitive impairments adversely affect social functioning (e.g., employment status) in patients with schizophrenia. Although pharmacological interventions have been suggested to provide some benefits on social cognition, little information is available on the comparative efficacy of pharmacotherapy. Thus, the aim of this planned systematic review and network meta-analysis is to perform a quantitative comparison of the effects of various psychotropic drugs, including supplements, on social cognition disturbances of schizophrenia.

**Methods:**

The literature search will be carried out using the PubMed, Embase, Cochrane Central Register of Controlled Trials, PsycINFO, ClinicalTrials.gov, and International Clinical Trials Registry Platform databases from inception onward. Randomized controlled trials that examined the efficacy of drugs in social cognitive disturbances will be included, based on the most recent studies and the broader literature than previously searched. This protocol defines *a priori* the methods that will be used for study selection, data collection, quality assessment, and statistical syntheses.

**Discussion:**

The findings this work are expected to help promote the development of better therapeutics of social cognitive impairments in schizophrenia and related psychiatric conditions.

**Systematic Review Registration:**

[www.crd.york.ac.uk/prospero], identifier [CRD42021293224].

## Introduction

Schizophrenia is one of the top causes of disability globally (GBD 2016 Disease and Injury Incidence and Prevalence Collaborators, 2017), with a prevalence of about 0.7% (Saha et al., 2005). Its symptoms include positive symptoms, negative symptoms, and cognitive impairments. While positive symptoms are well managed with antipsychotic drugs, negative symptoms and cognitive impairments usually remain unresolved (Haddad and Correll, 2018). In particular, 73% of patients with schizophrenia are reported to show cognitive disturbances, and up to 98% perform worse cognitively than would be predicted by their education level (Palmer et al., 1997; Keefe et al., 2005). These deficits apply to neurocognition, such as memory, attention, and executive function, as well as social cognition, such as emotion recognition, theory of mind (ToM), social perception, and attributional bias (Penn et al., 2008) (see Table 1).

**TABLE 1:** Key domains of social cognition [adapted from Pinkham et al. (2014)].

Domain	Definition	Examples of measures
Emotion recognition/processing	This domain is broadly defined as perceiving and using emotions. It subsumes three subdomains that represent both lower-level and higher-level processes. At a lower perceptual level is the first subdomain emotion perception/recognition (identifying and recognizing emotional displays from facial expressions and/or non-face cues such as voice) and at a higher level are the two subdomains of understanding emotions and managing emotions.	Bell Lysaker Emotion Recognition Task (BLERT), Diagnostic Analysis of Non-verbal Accuracy 2 (DANVA2), Face Emotion Discrimination Test (FEDT), Face Emotion Identification Test (FEIT), Penn Emotion Recognition Task (ER-40)
Theory of mind (ToM)	This domain is defined as the ability to represent the mental states of others, including the inference of intentions, dispositions, and/or beliefs. Theory of mind is also referred to as mentalizing, mental state attribution, or cognitive empathy.	Adult Faux Pas, Brune Picture Sequencing Task, Happe’s Stories, Reading the Mind in the Eyes Test, Silent Animations, The Awareness of Social Inference Test (TASIT), The Hinting Task, Visual Perspective Tasking Task, Social Cognition Screening Questionnaire (SCSQ)
Attributional bias/style	Attributional style describes the way in which individuals explain the causes, or make sense, of social events or interactions.	Ambiguous Intentions and Hostility Questionnaire (AIHQ), Internal, Personal, and Situational Attributions Questionnaire (IPSAQ), Intentionality Bias Task (IBT), Social Cognition Screening Questionnaire (SCSQ)
Social perception	Social perception refers to decoding and interpreting social cues in others. It includes social context processing and social knowledge, which can be defined as knowing social rules, roles, and goals (RRGs), utilizing those RRGs, and understanding how such RRGs may influence others’ behaviors.	Half Profile of Non-verbal Sensitivity (Half PONS), Interpersonal Perception Task (IPT-15), Relationships Across Domains (RAD), Social Attribution Task- Multiple Choice (SAT-MC), Situational Feature Recognition Test (SFRT)

Social cognition is defined as “the mental operations that underlie social interactions, including perceiving, interpreting, and generating responses to the intentions, dispositions, and behaviors of others” (Green and Leitman, 2008). Its impairment impedes social participation, e.g., interpersonal relationships, opportunities for education, and employment in patients with schizophrenia (Green et al., 2012). Social cognitive impairments have been reported to adversely affect social function and mediate the association between neurocognitive deficits and poor social consequences in patients with schizophrenia (Brekke et al., 2005; Halverson et al., 2019).

Among the domains of social cognition, ToM has been reported to reflect the degree of social cognitive impairment more distinctly (Rocca et al., 2016) and correlate more strongly with social functioning than other domains (Thibaudeau et al., 2021). Impairment of ToM is present from the onset of schizophrenia and correlates also with positive and negative symptoms, making it a cardinal component of social cognition (Ventura et al., 2015). Improvement of social cognitive impairments is important, since as many as 70% of these patients are currently unable to work (Marwaha and Johnson, 2004). Therefore, considerable effort has been directed to the development of treatment methods for social cognitive problems in schizophrenia (Yamada et al., 2021a,b, Yamada et al., 2022; Yamada and Sumiyoshi, 2022).

Several interventional methods are suggested to partially enhance social cognition or its sub-domains in patients with schizophrenia (Nijman et al., 2020; Yamada et al., 2021a; Yamada and Sumiyoshi, 2022). In a previous systematic review and meta-analysis (Nijman et al., 2020), psychosocial approaches, e.g., social-cognitive training (SCT), have been shown to improve several domains of social cognition, including emotion recognition and social perception. Among them, SCT provides non-pharmacological modality that particularly improve the domain of emotion recognition (Yeo et al., 2022).

Regarding pharmacological interventions, oxytocin (Bürkner et al., 2017), psychostimulants (e.g., modafinil) (Yamada et al., 2019), antipsychotic drugs (e.g., risperidone and olanzapine) (Gabay et al., 2015), and acetylcholinesterase inhibitors (e.g., donepezil) (Kishi et al., 2018) have been tested as candidate compounds (Fernández-Sotos et al., 2018). Results of meta-analyses indicate that intranasal oxytocin and atypical antipsychotic drugs may improve theory of mind (Bürkner et al., 2017) and emotion recognition (Gabay et al., 2015), respectively, while anti-dementia drugs (e.g., donepezil, galantamine, rivastigmine, and memantine) may elicit limited effects. On the other hand, emotional processing in healthy subjects becomes worse by diazepam, as indicated by meta-analysis (Haime et al., 2021). Therefore, additional research to promote the rational selection of compounds is desirable for facilitating the development of novel therapeutics to alleviate social cognitive deficits.

To our knowledge, no quantitative comparison has been made for the ability of psychotropic drugs to enhance social cognition in patients with schizophrenia. This initiative is important to better understand the neural basis of social cognitive impairments in schizophrenia and related psychotic disorders. Therefore, we prepared this study protocol for a planned systematic review and network meta-analysis of the literature on pharmacological interventions for social cognitive disturbances of schizophrenia.

## Methods

### Study design

We submitted this systematic review protocol for registration in the PROSPERO International Prospective Register of Systematic Reviews on November 27, 2021 (registration number is CRD42021293224). The protocol was prepared using the 2015 statement of Preferred Reporting Items for Systematic Reviews and Meta-Analysis Protocols (PRISMA-P) (Moher et al., 2015). The final review will be reported using the Preferred Reporting Items for Systematic Reviews and Meta-Analysis (PRISMA 2020) statement (Page et al., 2021).

### Search strategy

We will perform literature searches using the following electronic bibliographic databases from inception onward: PubMed, Embase, Cochrane Central Register of Controlled Trials (CENTRAL), PsycINFO, ClinicalTrials.gov, and International Clinical Trials Registry Platform (ICTRP). The search terms will comprise “schizophrenia,” “pharmacological intervention,” “social cognition,” and “randomized controlled trial” (see Supplementary Material 1). We will include search terms about the outcome, i.e., “social cognition,” because we need to solve the problem of too many candidate references and low specificity of the search.

### Eligibility criteria

Articles or clinical trial information must meet the following inclusion criteria:

(1) Randomized controlled trials including cluster and cross-over trials.
(2) Patients with schizophrenia or schizoaffective disorder based on F20–F29 of the International Statistical Classification of Diseases and Related Health Problems Version 10 (ICD-10) or schizophrenia spectrum and other psychotic disorders of the Diagnostic and Statistical Manual of Mental Disorders, 5th Edition (DSM-5), DSM-IV, or DSM- III.
(3) Drug interventions, e.g., hormone-related drugs, psychostimulants, anti-dementia drugs, antipsychotic drugs, antidepressant drugs, antibiotics, supplements, and placebos. With regard to the types of interventions, day-only or single doses will also be included in this review.
(4) At least 1 outcome used as an evaluation of social cognition (with only the 4 domains of emotion recognition, theory of mind, social perception, and attributional bias targeted).

Articles or clinical trial information with any of the following conditions will be excluded from the study:

(1) Reviews, editorials, protocols, or the reporting of data extracted from original articles.
(2) Single-arm trials or observational studies.
(3) Animal studies.
(4) *In vitro* studies.

Original articles or clinical trial information written in any languages will be included.

### Study selection

The YY will remove duplicate studies before the initial screening. We will divide 10 investigators (RK, HO, YH, YS, KT, SI, RS, TH, TU, and YY) into five groups of 2 people each. YY will divide all manuscripts into five batches, and each pair of investigators will oversee one-fifth of all manuscripts and independently perform the first round of screening based on the original title and abstract. The following steps will be taken to improve the accuracy of the initial screening. First, a manual on the inclusion and exclusion criteria will be prepared by YY and provided to all members. Second, at least 1 experienced systematic reviewer will be assigned to each group. Third, before starting the first screening, we will practice the screening of studies using the original title and abstract. At this stage, the Kappa statistics of each pair of investigators will be calculated to determine whether their ratings and agreement are reliable. Fourth, progress meetings with RO and YY will be held with each group after the screening has started, and all groups will be provided supplementary information about screening procedures as the need arises. Then, any study selected by at least 1 reviewer will be judged in the second round of screening based on the full text. In the second screening, 5 groups of 2 people each will independently evaluate the eligibility of the full text. To improve the accuracy of the second screening, we will follow the same practices and preparations as for the first screening. Finally, the senior reviewer (RO) will resolve any disagreements.

### Data extraction

We will divide six investigators (RK, HO, SI, TH, TU, and YY) into three groups of two people each. These groups will independently extract the following relevant information from the included studies: author, year of publication, country, trial design, participants’ demographics, details of the diagnosis, details of the intervention, control condition, duration of follow-up, and measurement tools. Additional outcomes will include measures of positive/negative symptoms, number of dropouts, and neurocognition. For measurements of social cognition, data will be extracted through outcomes relating to the following 4 domains; emotion recognition, theory of mind, social perception, and attributional bias to calculate the standardized mean difference for each intervention (Table 1). Using data from these domains, we will assess separately the effects of interventions on each of the four domains. Whether or not to conduct network meta-analysis for all domains will be carefully discussed within our study group from multiple perspectives, including the number of studies that could be included and the existence of closed loops (Salanti et al., 2008). With regard to the classification of drugs, information on individual compound will be extracted for each study. In the network meta-analysis, in parallel with the analysis for each compound, treatment effects will be tested according to the following classifications; hormone-related drugs, psychostimulants, anti-dementia drugs, antipsychotic drugs, antidepressant drugs, antibiotics, supplements, and placebos. To improve the accuracy of the data extraction, we will proceed as follows. First, a data extraction form and manual will be prepared by YY and provided to all members. Second, at least 1 experienced systematic reviewer will be assigned to each group. Third, before starting the data extraction, we will practice the data extraction using sample articles. Fourth, progress meetings with RO and YY will be held with each group after data extraction has started, and all groups will be provided supplementary information about data extraction as the need arises. The senior reviewer (RO) will resolve any disagreements. If it is not clear whether the measurement refers to social cognition, MN will ask the author(s) for additional information about the outcomes. For ongoing clinical trial information included in the second screening, MN will also ask the researcher(s) for detailed information. If MN does not receive a reply from the author(s), MN will repeat our inquiry up to three times with an interval of 1 week. If this approach is not successful or feasible, these studies will be excluded from the analysis.

There may be a certain number of studies that meet the inclusion criteria for systematic review, but lack the information on measures of social cognition used. The results of such studies will be included in the systematic review, but not in the meta-analysis. Instead, all studies included in the systematic review will be presented in a summary table.

### Quality assessments and risk of bias

Three groups of 2 people each (RK, HO, SI, TH, TU, and YY) will independently perform a quality assessment using the Cochrane Collaboration’s risk of bias tool 2 (Sterne et al., 2019), which assesses potential biases through signaling questions in the following domains: bias arising from the randomization process, bias due to deviations from intended interventions, bias due to missing outcome data, bias in measurement of the outcome, and bias in selection of the reported result. To improve the accuracy of the quality assessments, we will take the following steps. First, all members will attend a training course on quality assessment using the Cochrane Collaboration’s risk of bias tool 2, comprising self-study materials and 2 days of web-based exercise organized by Cochrane Japan. Second, at least 1 experienced systematic reviewer will be assigned to each group. Third, before starting the quality assessment, we will practice the quality assessment using sample articles. At this stage, the Kappa statistics of each pair of investigators will be calculated to determine whether their ratings and agreement are reliable. Fourth, progress meetings with RO and YY will be held in each group after the assessment has started, and all groups will be taught supplementary information about assessing qualities. The senior reviewer (RO) will reconcile any disagreements.

### Statistical analyses

The aim of the planned review is to generate comparisons among drugs in an attempt to determine which pharmacological interventions more effectively improve social cognition impairments in schizophrenia. Accordingly, studies will be integrated across a variety of pharmacological interventions if the measured outcomes are comparable and the data are available. In the case of a multi-arm study, a multivariate meta-analysis approach is used, considering the correlation of results within the same study (Jackson et al., 2011). If more than one scale is used for the same domain in the same test, the more frequently used scale, as indicated in Table 1, is adopted. The final decision on which test to adopt will be made prior to analyses through discussion within the research group, referring to the results of previous studies that have identified which tests are commonly used for the four domains of social cognition (Pinkham et al., 2014; Okano et al., 2021). Based on the assumption of transitivity, we will make direct/indirect comparisons between the various drug/compound interventions, assuming consistency that direct and indirect evidence are in agreement. On the other hand, the consistency will be carefully assessed using the following steps. First, based on the results of systematic reviews, heterogeneity between trials, which is one of major causes of inconsistency, will be assessed. Second, we will apply the consistency and inconsistency models to the trials included in the network meta-analysis, respectively, and compare their respective deviance information criterion (DIC) to assess overall consistency. In addition, the partial consistency of each loop will be also assessed by inconsistency factors (Veroniki et al., 2013). When heterogeneity of the meta-analysis is great, sensitivity analysis will be used to identify the causes of the increased heterogeneity, and subgroup analyses will be considered, excluding the studies that are responsible. If sufficient number of trials are available, we will consider performing analyses to correct for bias across trials, such as network meta-regression. All statistical analyses will be conducted within a Bayesian framework using R^1^ or Python^2^ with Open BUGS software^3^, JAGS^4^, or Stan^5^.

If comparable data are available, pairwise meta-analyses of each intervention will be performed as an exploratory analysis to explore the data. Significant heterogeneity is expected, so a random-effects model will be used. Forest plots will also be created to graphically depict the individual and pooled effect sizes.

Based on this principle, a network meta-analysis random effects model based on the standardized mean difference will be generated. All drugs for which data are available will be included in the model. The model will be based on a Bayesian framework, and vague priors will be used to ensure that the results are as close as possible to findings obtained from a frequentist approach. This will be implemented by setting the distributions with very broad precision. The network meta-analysis will generate pairwise comparisons between all drugs and the rankings of the drugs and will assess the probability that each drug is the best.

## Discussion

Our planned study will systematically review and analyze the comparative efficacy of pharmacological interventions for social cognitive impairments in schizophrenia.

The strength of this study is that it will examine a wide range of electronic bibliographic databases, and include the most recent articles written in any languages. In addition, the network meta-analysis approach will allow us to compare the effects on social cognition between psychotropic drugs that have not been compared in real clinical trials. Above all, this network meta-analysis will provide quantitative evidence about the effects of each class of psychotropic drugs on respective domain of social cognition. This may provide useful insights into which psychotropic drugs will be beneficial to patients with impairments in specific social cognitive domains.

However, the proposed systematic review and network meta-analysis will have some limitations. First, although the usual systematic review does not include search terms related to outcome, i.e., “social cognition,” we will include terms about it, because we need to solve the problem of too many candidate references and low specificity of the search. Second, the possible use of different scales to measure social cognition may cause heterogeneity across studies. Third, social cognition is divided into four domains, and, if we cannot obtain enough number of studies with pharmacological intervention from the literature for each domain, the significance of the network meta-analysis may be limited.

## Data availability statement

The original contributions presented in the study are included in the article/Supplementary material, further inquiries can be directed to the corresponding author.

## Author contributions

YY wrote the first draft of the manuscript. TS, RO, NW, and HT reviewed it and provided critical revisions. Other authors also revised the manuscript. All authors approved the final version of the manuscript.

## Footnotes

1
https://www.r-project.org

2
https://www.python.org/

3
https://www.openbugs.net

4
https://mcmc-jags.sourceforge.io

5
https://mc-stan.org
