# Supplementary material for: Pharmacological interventions for social cognitive impairments in schizophrenia: A protocol for a systematic review and network meta-analysis
Source: Front Psychol. 2022 Aug 3;13:878829. doi: 10.3389/fpsyg.2022.878829 (PMC9381750; doi:10.3389/fpsyg.2022.878829)
Supplement: Supplementary file 1 [file Data_Sheet_1.pdf]

Supplementary Material 1

Title
Pharmacological intervention for social cognitive impairments in schizophrenia: A protocol for systematic review and network meta-analysis

List
Search strategy for the following; Pharmacological intervention for social cognitive impairments in schizophrenia.
(1) Search strategy in PubMed
(2) Search strategy in Embase
(3) Search strategy in CENTRAL
(4) Search strategy in PsycInfo
(5) Search strategy in Clinical Trials.gov
(6) Search strategy in ICTRP

Databases
- PubMed
- Embase
- The Cochrane Central Register of Controlled Trials (CENTRAL) (The Cochrane Library)
- PsycInfo
- Clinical Trials.gov
- International Clinical Trials Registry Platform (ICTRP)

(1) PubMed

((("Schizophrenia"[Mesh] OR "Schizophrenia"[tw] OR "Schizophrenias"[tw] OR "Schizophrenic"[tw] OR "Schizophren*"[tw] OR "Dementia Praecox"[tw] OR "schizoaffective"[tw]) AND ("Drug Therapy"[Mesh] OR "Drug Therapy"[tw] OR "Drug Treatment"[tw] OR "pharmacotherapy"[tw] OR "pharmacotherap*"[tw] OR "pharma*"[tw] OR "drug"[tw] OR "drugs"[tw] OR "Oxytocin"[mesh] OR "Oxytocin"[tw] OR "Oxytocics"[Pharmacological Action] OR "Oxytocics"[mesh] OR "psychostimulant"[tw] OR "psychostimulants"[tw] OR "Central Nervous System Stimulants"[Mesh] OR "Central Nervous System Stimulants"[Pharmacological Action] OR "Central Nervous System Stimulants"[tw] OR "Central Nervous System Stimulant"[tw] OR "anti-dementia"[tw] OR "Nootropic Agents"[Mesh] OR "Nootropic Agents"[Pharmacological Action] OR "Nootropic Agents"[tw] OR "Nootropic Agent"[tw] OR "antipsychotics"[tw] OR "antipsychotic"[tw] OR "anti psychotics"[tw] OR "anti psychotic"[tw] OR "Antipsychotic Agents"[Mesh] OR "Antipsychotic Agents"[Pharmacological Action] OR "Schizophrenia/drug therapy"[Mesh] OR "Varenicline"[Mesh] OR "varenicline"[tiab] OR "Testosterone"[Mesh] OR "testosterone"[tiab] OR "Buprenorphine"[Mesh] OR "buprenorphine"[tiab] OR "Hydrocortisone"[Mesh] OR "hydrocortisone"[tiab] OR "Serotonin Uptake Inhibitors"[Mesh] OR "Serotonin Uptake Inhibitors"[Pharmacological Action] OR "Serotonin Uptake Inhibitors"[tiab] OR "Serotonin Uptake Inhibitor"[tiab] OR

"SSRI"[tiab] OR "SSRIs"[tiab] OR "Bumetanide"[Mesh] OR "bumetanide"[tiab] OR "Withania"[Mesh] OR "Withania"[tiab] OR "Psilocybin"[Mesh] OR "Psilocybin"[tiab] OR "Arginine Vasopressin"[Mesh] OR "Arginine Vasopressin"[tiab] OR "Androstadienes"[Mesh] OR "androstadienone"[tiab] OR "Valproic Acid"[Mesh] OR "Valproic Acid"[tiab] OR "divalproex sodium"[tiab] OR "Tryptophan"[Mesh] OR "Tryptophan"[tiab]) AND ("social cognitive impairments"[tiab] OR "social cognitive impairment"[tiab] OR "emotion perception"'[tiab] OR "social perception"[tiab] OR "theory of mind"[tiab] OR "attributional bias"[tiab] **OR "social cues"'[tiab] OR "social cue"[tiab] OR "emotional cues"'[tiab] OR "emotional cue"[tiab] OR "affect recognition"[tiab] OR "approach behavior"[tiab] OR "approach behaviors"[tiab] OR "approach behaviour"[tiab] OR "approach behaviours"[tiab] OR "cognitive processing"[tiab] OR "cognitive symptom"[tiab] OR "cognitive symptoms"[tiab] OR "emotion recognition"[tiab] OR "emotional expression"[tiab] OR "emotional expressions"[tiab] OR "emotional face perception"[tiab] OR "emotional face processing"[tiab] OR "emotional face"[tiab] OR "emotional faces"[tiab] OR "emotional processing"[tiab] OR "emotional reactivity"[tiab] OR "emotional stimuli"[tiab] OR "emotional stimulus"[tiab] OR "facial expressios"[tiab] OR "facial expressions"[tiab] OR "mentalizing"[tiab] OR "social face processing"[tiab] OR "social perception"[tiab] OR "socioaffective stimuli"[tiab] OR "socioaffective stimulus"[tiab] OR "socio affective stimuli"[tiab] OR "socio affective stimulus"[tiab]** OR "emotion processing"[tiab] OR "emotional empathy"[tiab] OR "emotional functioning"[tiab] OR "emphatic concern"[tiab] OR "emphatic concerns"[tiab] OR "mind reading"[tiab] OR "recognition memory"[tiab] OR "social memories"[tiab] OR "social memory"[tiab] OR "vicarious emotion"[tiab] OR "vicarious emotions"[tiab] *OR (("Conflict, Psychological"[mesh] OR "Empathy"[mesh] OR "Emotions"[mesh] OR "Psychomotor Performance"[mesh] OR "Facial Expression"[mesh] OR "Violence"[mesh] OR "Temperament"[mesh]) AND "Social Behavior"[mesh])* OR "emotional cognitions"[tiab] OR "emotional cognition"[tiab] OR ("Cognition"[mesh:noexp] AND "Emotions"[mesh:noexp])) AND (randomized controlled trial[pt] OR controlled clinical trial[pt] OR randomized[tiab] OR placebo[tiab] OR drug therapy[sh] OR randomly[tiab] OR trial[tiab] OR groups[tiab]) NOT (animals [mh] NOT humans [mh])) **OR** (("Oxytocin"[mesh] OR "Oxytocin"[ti] OR "Oxytocics"[Pharmacological Action] OR "Oxytocics"[mesh] OR "psychostimulant"[ti] OR "psychostimulants"[ti] OR "Central Nervous System Stimulants"[mesh] OR "Central Nervous System Stimulants"[Pharmacological Action] OR "Central Nervous System Stimulants"[ti] OR "Central Nervous System Stimulant"[ti] OR "anti-dementia"[ti] OR "Nootropic Agents"[mesh] OR "Nootropic Agents"[Pharmacological Action] OR "Nootropic Agents"[ti] OR "Nootropic Agent"[ti] OR "antipsychotics"[ti] OR "antipsychotic"[ti] OR "anti psychotics"[ti] OR "anti psychotic"[ti] OR "Antipsychotic Agents"[mesh] OR "Antipsychotic Agents"[Pharmacological Action] OR "Schizophrenia/drug therapy"[mesh] OR "Varenicline"[Mesh] OR "varenicline"[tiab] OR "Testosterone"[Mesh] OR "testosterone"[tiab] OR "Buprenorphine"[Mesh] OR "buprenorphine"[tiab] OR "Hydrocortisone"[Mesh] OR "hydrocortisone"[tiab] OR "Serotonin Uptake Inhibitors"[Mesh] OR "Serotonin Uptake Inhibitors"[Pharmacological Action] OR "Serotonin Uptake Inhibitors"[tiab] OR "Serotonin Uptake Inhibitor"[tiab] OR

"SSRI"[tiab] OR "SSRIs"[tiab] OR "Bumetanide"[Mesh] OR "bumetanide"[tiab] OR "Withania"[Mesh] OR "Withania"[tiab] OR "Psilocybin"[Mesh] OR "Psilocybin"[tiab] OR "Arginine Vasopressin"[Mesh] OR "Arginine Vasopressin"[tiab] OR "Androstadienes"[Mesh] OR "androstadienone"[tiab] OR "Valproic Acid"[Mesh] OR "Valproic Acid"[tiab] OR "divalproex sodium"[tiab] OR "Tryptophan"[Mesh] OR "Tryptophan"[tiab]) AND ("social cognitive impairments"[tiab] OR "social cognitive impairment"[tiab] OR "social cognition"[tiab] OR "emotion perception"'[tiab] OR "social perception"[tiab] OR "theory of mind"[tiab] OR "attributional bias"[tiab] **OR "social cues"'[tiab] OR "social cue"[tiab] OR "emotional cues"'[tiab] OR "emotional cue"[tiab] OR "affect recognition"[tiab] OR "approach behavior"[tiab] OR "approach behaviors"[tiab] OR "approach behaviour"[tiab] OR "approach behaviours"[tiab] OR "cognitive processing"[tiab] OR "cognitive symptom"[tiab] OR "cognitive symptoms"[tiab] OR "emotion recognition"[tiab] OR "emotional expression"[tiab] OR "emotional expressions"[tiab] OR "emotional face perception"[tiab] OR "emotional face processing"[tiab] OR "emotional face"[tiab] OR "emotional faces"[tiab] OR "emotional processing"[tiab] OR "emotional reactivity"[tiab] OR "emotional stimuli"[tiab] OR "emotional stimulus"[tiab] OR "facial expressios"[tiab] OR "facial expressions"[tiab] OR "mentalizing"[tiab] OR "social face processing"[tiab] OR "social perception"[tiab] OR "socioaffective stimuli"[tiab] OR "socioaffective stimulus"[tiab] OR "socio affective stimuli"[tiab] OR "socio affective stimulus"[tiab]** OR "emotion processing"[tiab] OR "emotional empathy"[tiab] OR "emotional functioning"[tiab] OR "emphatic concern"[tiab] OR "emphatic concerns"[tiab] OR "mind reading"[tiab] OR "recognition memory"[tiab] OR "social memories"[tiab] OR "social memory"[tiab] OR "vicarious emotion"[tiab] OR "vicarious emotions"[tiab] *OR (("Conflict, Psychological"[mesh] OR "Empathy"[mesh] OR "Emotions"[mesh] OR "Psychomotor Performance"[mesh] OR "Facial Expression"[mesh] OR "Violence"[mesh] OR "Temperament"[mesh]) AND "Social Behavior"[mesh])* OR "emotional cognitions"[tiab] OR "emotional cognition"[tiab] OR ("Cognition"[mesh:noexp] AND "Emotions"[mesh:noexp])) AND (randomized controlled trial[pt] OR controlled clinical trial[pt] OR randomized[tiab] OR placebo[tiab] OR drug therapy[sh] OR randomly[tiab] OR trial[tiab] OR groups[tiab]) NOT (animals [mh] NOT humans [mh])) **OR** (("pharmacology"[Subheading] OR "drug therapy"[Subheading]) AND ("social cognitive impairments"[ti] OR "social cognitive impairment"[ti] OR "social cognition"[ti] OR "emotion perception"'[ti] OR "social perception"[ti] OR "theory of mind"[ti] OR "attributional bias"[ti] **OR "social cues"'[ti] OR "social cue"[ti] OR "emotional cues"'[ti] OR "emotional cue"[ti] OR "affect recognition"[ti] OR "approach behavior"[ti] OR "approach behaviors"[ti] OR "approach behaviour"[ti] OR "approach behaviours"[ti] OR "cognitive processing"[ti] OR "cognitive symptom"[ti] OR "cognitive symptoms"[ti] OR "emotion recognition"[ti] OR "emotional expression"[ti] OR "emotional expressions"[ti] OR "emotional face perception"[ti] OR "emotional face processing"[ti] OR "emotional face"[ti] OR "emotional faces"[ti] OR "emotional processing"[ti] OR "emotional reactivity"[ti] OR "emotional stimuli"[ti] OR "emotional stimulus"[ti] OR "facial expressios"[ti] OR "facial expressions"[ti] OR "mentalizing"[ti] OR "social face processing"[ti] OR "social perception"[ti] OR "socioaffective stimuli"[ti] OR "socioaffective stimulus"[ti] OR**

"socio affective stimuli"[ti] OR "socio affective stimulus"[ti] OR "emotion processing"[ti] OR "emotional empathy"[ti] OR "emotional functioning"[ti] OR "emphatic concern"[ti] OR "emphatic concerns"[ti] OR "mind reading"[ti] OR "recognition memory"[ti] OR "social memories"[ti] OR "social memory"[ti] OR "vicarious emotion"[ti] OR "vicarious emotions"[ti]) AND (randomized controlled trial[pt] OR controlled clinical trial[pt] OR randomized[tiab] OR placebo[tiab] OR drug therapy[sh] OR randomly[tiab] OR trial[tiab] OR groups[tiab]) NOT (animals [mh] NOT humans [mh])))

(2) Embase

(((exp *"Schizophrenia"/ OR "Schizophrenia".ti,ab OR "Schizophrenias".ti,ab OR "Schizophrenic".ti,ab OR "Schizophren*".ti,ab OR "Dementia Praecox".ti,ab OR "schizoaffective".ti,ab) AND (exp *"Drug Therapy"/ OR "Drug Therapy".ti,ab OR "Drug Treatment".ti,ab OR "pharmacotherapy".ti,ab OR "pharmacotherap*".ti,ab OR "pharma*".ti,ab OR "drug".ti,ab OR "drugs".ti,ab OR "Oxytocin"/ OR "Oxytocin".ti,ab OR "Oxytocic agent"/ OR "psychostimulant".ti,ab OR "psychostimulants".ti,ab OR exp *"psychostimulant agent"/ OR exp *"Central Stimulant agent"/ OR "Central Nervous System Stimulants".ti,ab OR "Central Nervous System Stimulant".ti,ab OR "anti-dementia".ti,ab OR exp *"Nootropic Agent"/ OR "Nootropic Agents".ti,ab OR "Nootropic Agent".ti,ab OR "antipsychotics".ti,ab OR "antipsychotic".ti,ab OR "anti psychotics".ti,ab OR "anti psychotic".ti,ab OR exp *"Neuroleptic Agent"/ OR exp *"Schizophrenia"/dt OR "Varenicline"/ OR "varenicline".ti,ab OR "Testosterone"/ OR "testosterone".ti,ab OR "Buprenorphine"/ OR "buprenorphine".ti,ab OR "Hydrocortisone"/ OR "hydrocortisone".ti,ab OR exp *"Serotonin Uptake Inhibitor"/ OR "Serotonin Uptake Inhibitors".ti,ab OR "Serotonin Uptake Inhibitor".ti,ab OR "SSRI".ti,ab OR "SSRIs".ti,ab OR "Bumetanide"/ OR "bumetanide".ti,ab OR "Withania"/ OR "Withania".ti,ab OR "Psilocybin"/ OR "Psilocybin".ti,ab OR "Arginine Vasopressin"/ OR "Arginine Vasopressin".ti,ab OR exp *"androstane derivative"/ OR "androstadienone".ti,ab OR "Valproic Acid"/ OR "Valproic Acid".ti,ab OR "divalproex sodium".ti,ab OR "Tryptophan"/ OR "Tryptophan".ti,ab) AND ("social cognitive impairments".ti,ab OR "social cognitive impairment".ti,ab OR "emotion perception"'.ti,ab OR "social perception".ti,ab OR "theory of mind".ti,ab OR "attributional bias".ti,ab **OR "social cues"'.ti,ab OR "social cue".ti,ab OR "emotional cues"'.ti,ab OR "emotional cue".ti,ab OR "affect recognition".ti,ab OR "approach behavior".ti,ab OR "approach behaviors".ti,ab OR "approach behaviour".ti,ab OR "approach behaviours".ti,ab OR "cognitive processing".ti,ab OR "cognitive symptom".ti,ab OR "cognitive symptoms".ti,ab OR "emotion recognition".ti,ab OR "emotional expression".ti,ab OR "emotional expressions".ti,ab OR "emotional face perception".ti,ab OR "emotional face processing".ti,ab OR "emotional face".ti,ab OR "emotional faces".ti,ab OR "emotional processing".ti,ab OR "emotional reactivity".ti,ab OR "emotional stimuli".ti,ab OR "emotional stimulus".ti,ab OR "facial expressios".ti,ab OR "facial expressions".ti,ab OR "mentalizing".ti,ab OR "social face processing".ti,ab OR "social perception".ti,ab OR "socioaffective stimuli".ti,ab OR "socioaffective stimulus".ti,ab OR "socio affective stimuli".ti,ab OR "socio affective**

stimulus".ti,ab OR "emotion processing".ti,ab OR "emotional empathy".ti,ab OR "emotional functioning".ti,ab OR "emphatic concern".ti,ab OR "emphatic concerns".ti,ab OR "mind reading".ti,ab OR "recognition memory".ti,ab OR "social memories".ti,ab OR "social memory".ti,ab OR "vicarious emotion".ti,ab OR "vicarious emotions".ti,ab _OR (("Conflict"/ OR exp *"Empathy"/ OR exp *"Emotion"/ OR exp *"Psychomotor Performance"/ OR "Facial Expression"/ OR exp *"Violence"/ OR exp *"Temperament"/) AND exp *"Social Behavior"/)_ OR "emotional cognitions".ti,ab OR "emotional cognition".ti,ab OR ("Cognition"/ AND "Emotion"/)) AND (exp *"randomized controlled trial"/ OR exp *"controlled clinical trial"/ OR randomized.ti,ab OR placebo.ti,ab OR randomly.ti,ab OR trial.ti,ab OR groups.ti,ab) NOT (exp *animals/ NOT exp *humans/)) **OR** (("Oxytocin"/ OR "Oxytocin".ti,ab OR "Oxytocic agent"/ OR "psychostimulant".ti,ab OR "psychostimulants".ti,ab OR exp *"psychostimulant agent"/ OR exp *"Central Stimulant agent"/ OR "Central Nervous System Stimulants".ti,ab OR "Central Nervous System Stimulant".ti,ab OR "anti-dementia".ti,ab OR exp *"Nootropic Agent"/ OR "Nootropic Agents".ti,ab OR "Nootropic Agent".ti,ab OR "antipsychotics".ti,ab OR "antipsychotic".ti,ab OR "anti psychotics".ti,ab OR "anti psychotic".ti,ab OR exp *"Neuroleptic Agent"/ OR exp *"Schizophrenia"/dt OR "Varenicline"/ OR "varenicline".ti,ab OR "Testosterone"/ OR "testosterone".ti,ab OR "Buprenorphine"/ OR "buprenorphine".ti,ab OR "Hydrocortisone"/ OR "hydrocortisone".ti,ab OR exp *"Serotonin Uptake Inhibitor"/ OR "Serotonin Uptake Inhibitors".ti,ab OR "Serotonin Uptake Inhibitor".ti,ab OR "SSRI".ti,ab OR "SSRIs".ti,ab OR "Bumetanide"/ OR "bumetanide".ti,ab OR "Withania"/ OR "Withania".ti,ab OR "Psilocybin"/ OR "Psilocybin".ti,ab OR "Arginine Vasopressin"/ OR "Arginine Vasopressin".ti,ab OR exp *"androstane derivative"/ OR "androstadienone".ti,ab OR "Valproic Acid"/ OR "Valproic Acid".ti,ab OR "divalproex sodium".ti,ab OR "Tryptophan"/ OR "Tryptophan".ti,ab) AND ("social cognitive impairments".ti,ab OR "social cognitive impairment".ti,ab OR "emotion perception"'.ti,ab OR "social perception".ti,ab OR "theory of mind".ti,ab OR "attributional bias".ti,ab **OR "social cues"'.ti,ab OR "social cue".ti,ab OR "emotional cues"'.ti,ab OR "emotional cue".ti,ab OR "affect recognition".ti,ab OR "approach behavior".ti,ab OR "approach behaviors".ti,ab OR "approach behaviour".ti,ab OR "approach behaviours".ti,ab OR "cognitive processing".ti,ab OR "cognitive symptom".ti,ab OR "cognitive symptoms".ti,ab OR "emotion recognition".ti,ab OR "emotional expression".ti,ab OR "emotional expressions".ti,ab OR "emotional face perception".ti,ab OR "emotional face processing".ti,ab OR "emotional face".ti,ab OR "emotional faces".ti,ab OR "emotional processing".ti,ab OR "emotional reactivity".ti,ab OR "emotional stimuli".ti,ab OR "emotional stimulus".ti,ab OR "facial expressios".ti,ab OR "facial expressions".ti,ab OR "mentalizing".ti,ab OR "social face processing".ti,ab OR "social perception".ti,ab OR "socioaffective stimuli".ti,ab OR "socioaffective stimulus".ti,ab OR "socio affective stimuli".ti,ab OR "socio affective stimulus".ti,ab** OR "emotion processing".ti,ab OR "emotional empathy".ti,ab OR "emotional functioning".ti,ab OR "emphatic concern".ti,ab OR "emphatic concerns".ti,ab OR "mind reading".ti,ab OR "recognition memory".ti,ab OR "social memories".ti,ab OR "social memory".ti,ab OR "vicarious emotion".ti,ab OR "vicarious emotions".ti,ab _OR (("Conflict"/ OR exp *"Empathy"/ OR exp *"Emotion"/ OR exp *"Psychomotor Performance"/ OR "Facial Expression"/ OR exp *"Violence"/ OR exp_

*"Temperament"/) AND exp *"Social Behavior"/)* OR "emotional cognitions".ti,ab OR "emotional cognition".ti,ab OR ("Cognition"/ AND "Emotion"/)) AND (exp *"randomized controlled trial"/ OR exp *"controlled clinical trial"/ OR randomized.ti,ab OR placebo.ti,ab OR randomly.ti,ab OR trial.ti,ab OR groups.ti,ab) NOT (exp *animals/ NOT exp *humans/)) **OR** (("pd".fs OR "dt".fs) AND ("social cognitive impairments".ti OR "social cognitive impairment".ti OR "social cognition".ti OR "emotion perception"'.ti OR "social perception".ti OR "theory of mind".ti OR "attributional bias".ti **OR "social cues"'.ti OR "social cue".ti OR "emotional cues"'.ti OR "emotional cue".ti OR "affect recognition".ti OR "approach behavior".ti OR "approach behaviors".ti OR "approach behaviour".ti OR "approach behaviours".ti OR "cognitive processing".ti OR "cognitive symptom".ti OR "cognitive symptoms".ti OR "emotion recognition".ti OR "emotional expression".ti OR "emotional expressions".ti OR "emotional face perception".ti OR "emotional face processing".ti OR "emotional face".ti OR "emotional faces".ti OR "emotional processing".ti OR "emotional reactivity".ti OR "emotional stimuli".ti OR "emotional stimulus".ti OR "facial expressios".ti OR "facial expressions".ti OR "mentalizing".ti OR "social face processing".ti OR "social perception".ti OR "socioaffective stimuli".ti OR "socioaffective stimulus".ti OR "socio affective stimuli".ti OR "socio affective stimulus".ti** OR "emotion processing".ti OR "emotional empathy".ti OR "emotional functioning".ti OR "emphatic concern".ti OR "emphatic concerns".ti OR "mind reading".ti OR "recognition memory".ti OR "social memories".ti OR "social memory".ti OR "vicarious emotion".ti OR "vicarious emotions".ti) AND (exp *"randomized controlled trial"/ OR exp *"controlled clinical trial"/ OR randomized.ti,ab OR placebo.ti,ab OR randomly.ti,ab OR trial.ti,ab OR groups.ti,ab) NOT (exp *animals/ NOT exp *humans/))**)**

(3) CENTRAL

((("Schizophrenia" OR "Schizophrenia" OR "Schizophrenias" OR "Schizophrenic" OR "Schizophren*" OR "Dementia Praecox" OR "schizoaffective"):ti,ab,kw AND ("Drug Therapy" OR "Drug Therapy" OR "Drug Treatment" OR "pharmacotherapy" OR "pharmacotherap*" OR "pharma*" OR "drug" OR "drugs" OR "Oxytocin" OR "Oxytocin" OR "Oxytocic agent" OR "psychostimulant" OR "psychostimulants" OR "psychostimulant agent" OR "Central Stimulant agent" OR "Central Nervous System Stimulants" OR "Central Nervous System Stimulant" OR "anti dementia" OR "Nootropic Agent" OR "Nootropic Agents" OR "Nootropic Agent" OR "antipsychotics" OR "antipsychotic" OR "anti psychotics" OR "anti psychotic" OR "Neuroleptic Agent" OR "Varenicline" OR "varenicline" OR "Testosterone" OR "testosterone" OR "Buprenorphine" OR "buprenorphine" OR "Hydrocortisone" OR "hydrocortisone" OR "Serotonin Uptake Inhibitor" OR "Serotonin Uptake Inhibitors" OR "Serotonin Uptake Inhibitor" OR "SSRI" OR "SSRIs" OR "Bumetanide" OR "bumetanide" OR "Withania" OR "Withania" OR "Psilocybin" OR "Psilocybin" OR "Arginine Vasopressin" OR "Arginine Vasopressin" OR "androstane derivative" OR "androstadienone" OR "Valproic Acid" OR "Valproic Acid" OR "divalproex sodium" OR "Tryptophan" OR "Tryptophan"):ti,ab,kw AND ("social cognitive impairments" OR "social cognitive impairment" OR "emotion perception"' OR "social perception" OR "theory of mind"

OR "attributional bias" **OR "social cues"' OR "social cue" OR "emotional cues"' OR "emotional cue" OR "affect recognition" OR "approach behavior" OR "approach behaviors" OR "approach behaviour" OR "approach behaviours" OR "cognitive processing" OR "cognitive symptom" OR "cognitive symptoms" OR "emotion recognition" OR "emotional expression" OR "emotional expressions" OR "emotional face perception" OR "emotional face processing" OR "emotional face" OR "emotional faces" OR "emotional processing" OR "emotional reactivity" OR "emotional stimuli" OR "emotional stimulus" OR "facial expressios" OR "facial expressions" OR "mentalizing" OR "social face processing" OR "social perception" OR "socioaffective stimuli" OR "socioaffective stimulus" OR "socio affective stimuli" OR "socio affective stimulus"** OR "emotion processing" OR "emotional empathy" OR "emotional functioning" OR "emphatic concern" OR "emphatic concerns" OR "mind reading" OR "recognition memory" OR "social memories" OR "social memory" OR "vicarious emotion" OR "vicarious emotions" *OR (("Conflict" OR "Empathy" OR "Emotion" OR "Psychomotor Performance" OR "Facial Expression" OR "Violence" OR "Temperament") AND "Social Behavior")* OR "emotional cognitions" OR "emotional cognition" OR ("Cognition" AND "Emotion")):ti,ab,kw) **OR** (("Oxytocin" OR "Oxytocin" OR "Oxytocic agent" OR "psychostimulant" OR "psychostimulants" OR "psychostimulant agent" OR "Central Stimulant agent" OR "Central Nervous System Stimulants" OR "Central Nervous System Stimulant" OR "anti dementia" OR "Nootropic Agent" OR "Nootropic Agents" OR "Nootropic Agent" OR "antipsychotics" OR "antipsychotic" OR "anti psychotics" OR "anti psychotic" OR "Neuroleptic Agent" OR "Varenicline" OR "varenicline" OR "Testosterone" OR "testosterone" OR "Buprenorphine" OR "buprenorphine" OR "Hydrocortisone" OR "hydrocortisone" OR "Serotonin Uptake Inhibitor" OR "Serotonin Uptake Inhibitors" OR "Serotonin Uptake Inhibitor" OR "SSRI" OR "SSRIs" OR "Bumetanide" OR "bumetanide" OR "Withania" OR "Withania" OR "Psilocybin" OR "Psilocybin" OR "Arginine Vasopressin" OR "Arginine Vasopressin" OR "androstane derivative" OR "androstadienone" OR "Valproic Acid" OR "Valproic Acid" OR "divalproex sodium" OR "Tryptophan" OR "Tryptophan"):ti,ab,kw AND ("social cognitive impairments" OR "social cognitive impairment" OR "emotion perception"' OR "social perception" OR "theory of mind" OR "attributional bias" **OR "social cues"' OR "social cue" OR "emotional cues"' OR "emotional cue" OR "affect recognition" OR "approach behavior" OR "approach behaviors" OR "approach behaviour" OR "approach behaviours" OR "cognitive processing" OR "cognitive symptom" OR "cognitive symptoms" OR "emotion recognition" OR "emotional expression" OR "emotional expressions" OR "emotional face perception" OR "emotional face processing" OR "emotional face" OR "emotional faces" OR "emotional processing" OR "emotional reactivity" OR "emotional stimuli" OR "emotional stimulus" OR "facial expressios" OR "facial expressions" OR "mentalizing" OR "social face processing" OR "social perception" OR "socioaffective stimuli" OR "socioaffective stimulus" OR "socio affective stimuli" OR "socio affective stimulus"** OR "emotion processing" OR "emotional empathy" OR "emotional functioning" OR "emphatic concern" OR "emphatic concerns" OR "mind reading" OR "recognition memory" OR "social memories" OR "social memory" OR "vicarious emotion" OR "vicarious emotions" *OR (("Conflict" OR "Empathy" OR "Emotion" OR*

"Psychomotor Performance" OR "Facial Expression" OR "Violence" OR "Temperament") AND "Social Behavior") OR "emotional cognitions" OR "emotional cognition" OR ("Cognition" AND "Emotion")):ti,ab,kw))

(4) PsycINFO

TX((("Schizophrenia" OR "Schizophrenia" OR "Schizophrenias" OR "Schizophrenic" OR "Schizophren*" OR "Dementia Praecox" OR "schizoaffective") AND ("Drug Therapy" OR "Drug Therapy" OR "Drug Treatment" OR "pharmacotherapy" OR "pharmacotherap*" OR "pharma*" OR "drug" OR "drugs" OR "Oxytocin" OR "Oxytocin" OR "Oxytocic agent" OR "psychostimulant" OR "psychostimulants" OR "psychostimulant agent" OR "Central Stimulant agent" OR "Central Nervous System Stimulants" OR "Central Nervous System Stimulant" OR "anti dementia" OR "Nootropic Agent" OR "Nootropic Agents" OR "Nootropic Agent" OR "antipsychotics" OR "antipsychotic" OR "anti psychotics" OR "anti psychotic" OR "Neuroleptic Agent" OR "Varenicline" OR "varenicline" OR "Testosterone" OR "testosterone" OR "Buprenorphine" OR "buprenorphine" OR "Hydrocortisone" OR "hydrocortisone" OR "Serotonin Uptake Inhibitor" OR "Serotonin Uptake Inhibitors" OR "Serotonin Uptake Inhibitor" OR "SSRI" OR "SSRIs" OR "Bumetanide" OR "bumetanide" OR "Withania" OR "Withania" OR "Psilocybin" OR "Psilocybin" OR "Arginine Vasopressin" OR "Arginine Vasopressin" OR "androstane derivative" OR "androstadienone" OR "Valproic Acid" OR "Valproic Acid" OR "divalproex sodium" OR "Tryptophan" OR "Tryptophan") AND ("social cognitive impairments" OR "social cognitive impairment" OR "emotion perception"' OR "social perception" OR "theory of mind" OR "attributional bias" OR "social cues"' OR "social cue" OR "emotional cues"' OR "emotional cue" OR "affect recognition" OR "approach behavior" OR "approach behaviors" OR "approach behaviour" OR "approach behaviours" OR "cognitive processing" OR "cognitive symptom" OR "cognitive symptoms" OR "emotion recognition" OR "emotional expression" OR "emotional expressions" OR "emotional face perception" OR "emotional face processing" OR "emotional face" OR "emotional faces" OR "emotional processing" OR "emotional reactivity" OR "emotional stimuli" OR "emotional stimulus" OR "facial expressios" OR "facial expressions" OR "mentalizing" OR "social face processing" OR "social perception" OR "socioaffective stimuli" OR "socioaffective stimulus" OR "socio affective stimuli" OR "socio affective stimulus" OR "emotion processing" OR "emotional empathy" OR "emotional functioning" OR "emphatic concern" OR "emphatic concerns" OR "mind reading" OR "recognition memory" OR "social memories" OR "social memory" OR "vicarious emotion" OR "vicarious emotions" OR (("Conflict" OR "Empathy" OR "Emotion" OR "Psychomotor Performance" OR "Facial Expression" OR "Violence" OR "Temperament") AND "Social Behavior") OR "emotional cognitions" OR "emotional cognition" OR ("Cognition" AND "Emotion"))) OR (("Oxytocin" OR "Oxytocin" OR "Oxytocic agent" OR "psychostimulant" OR "psychostimulants" OR "psychostimulant agent" OR "Central Stimulant agent" OR "Central Nervous System Stimulants" OR "Central Nervous System Stimulant" OR "anti dementia" OR "Nootropic Agent" OR "Nootropic Agents" OR "Nootropic Agent" OR "antipsychotics" OR "antipsychotic" OR "anti psychotics" OR "anti psychotic" OR "Neuroleptic Agent" OR "Varenicline" OR "varenicline" OR "Testosterone" OR "testosterone" OR "Buprenorphine" OR

"buprenorphine" OR "Hydrocortisone" OR "hydrocortisone" OR "Serotonin Uptake Inhibitor" OR "Serotonin Uptake Inhibitors" OR "Serotonin Uptake Inhibitor" OR "SSRI" OR "SSRIs" OR "Bumetanide" OR "bumetanide" OR "Withania" OR "Withania" OR "Psilocybin" OR "Psilocybin" OR "Arginine Vasopressin" OR "Arginine Vasopressin" OR "androstane derivative" OR "androstadienone" OR "Valproic Acid" OR "Valproic Acid" OR "divalproex sodium" OR "Tryptophan" OR "Tryptophan") AND ("social cognitive impairments" OR "social cognitive impairment" OR "emotion perception"' OR "social perception" OR "theory of mind" OR "attributional bias" OR "social cues"' OR "social cue" OR "emotional cues"' OR "emotional cue" OR "affect recognition" OR "approach behavior" OR "approach behaviors" OR "approach behaviour" OR "approach behaviours" OR "cognitive processing" OR "cognitive symptom" OR "cognitive symptoms" OR "emotion recognition" OR "emotional expression" OR "emotional expressions" OR "emotional face perception" OR "emotional face processing" OR "emotional face" OR "emotional faces" OR "emotional processing" OR "emotional reactivity" OR "emotional stimuli" OR "emotional stimulus" OR "facial expressios" OR "facial expressions" OR "mentalizing" OR "social face processing" OR "social perception" OR "socioaffective stimuli" OR "socioaffective stimulus" OR "socio affective stimuli" OR "socio affective stimulus" OR "emotion processing" OR "emotional empathy" OR "emotional functioning" OR "emphatic concern" OR "emphatic concerns" OR "mind reading" OR "recognition memory" OR "social memories" OR "social memory" OR "vicarious emotion" OR "vicarious emotions" OR (("Conflict" OR "Empathy" OR "Emotion" OR "Psychomotor Performance" OR "Facial Expression" OR "Violence" OR "Temperament") AND "Social Behavior") OR "emotional cognitions" OR "emotional cognition" OR ("Cognition" AND "Emotion")))) AND (DE "Clinical Trials" OR DE "Randomized Controlled Trials" OR TX("randomized controlled trial" OR "controlled clinical trial" OR randomized OR placebo OR randomly OR trial OR groups)) NOT TI("veterinary" OR "rabbit" OR "rabbits" OR "animal" OR "animals" OR "mouse" OR "mice" OR "rodent" OR "rodents" OR "rat" OR "rats" OR "pig" OR "pigs" OR "porcine" OR "horse" OR "horses" OR "equine" OR "cow" OR "cows" OR "bovine" OR "goat" OR "goats" OR "sheep" OR "ovine" OR "canine" OR "dog" OR "dogs" OR "feline" OR "cat" OR "cats")

(5) Clinical Trials.gov
http://clinicaltrials.gov/

Condition/disease

Schizophrenia OR Schizophrenic OR Schizophren* OR Dementia Praecox OR schizoaffective

Intervention

Drug OR Drugs OR pharmacotherapy OR Oxytocin OR psychostimulant OR Varenicline OR Testosterone OR Buprenorphine OR Hydrocortisone OR SSRI OR bumetanide OR Withania OR Psilocybin OR Arginine Vasopressin OR androstadienone

OR Valproic Acid OR divalproex sodium OR Tryptophan

Other terms

social cognitive impairment OR emotion perception OR social perception OR theory of mind OR attributional bias OR social cue OR emotional cue OR affect recognition OR approach behavior OR cognitive processing OR cognitive symptom

(6) International Clinical Trials Registry Platform (ICTRP)
https://ictrptest.azurewebsites.net/Default.aspx

Condition/disease

Schizophrenia AND social cognitive impairment

Intervention

Drug OR Drugs OR pharmacotherapy
